# Swimming Behavior of *Percocypris pingi* in the Wake of D-Shaped Obstacles: A Comparative Study of Single- and Dual-Fish Swimming in Complex Hydrodynamic Environments

**DOI:** 10.3390/biomimetics10110749

**Published:** 2025-11-06

**Authors:** Lijian Ouyang, Qihao Meng, Qin Zhao, Liang Yu, Yike Li, Zebin Zhang, Li Tian, Zhiyuan Yang, Jiabin Lu, Weiwei Yao

**Affiliations:** 1 College of Ecological Engineering, Guizhou University of Engineering Science, Bijie 551700, China; oylj@gues.edu.cn (L.O.);; 2 State Key Laboratory of Hydraulics and Mountain River Engineering, Sichuan University, Chengdu 610065, China; 3 CHN Energy Dadu River Gongzui Power Generation Co., Ltd., Leshan 614000, China

**Keywords:** Kármán vortex street, group swimming, hydrodynamics, wake region, flow interference, fish swimming behavior

## Abstract

The changes in water flow caused by hydropower projects and river diversions have had a profound impact on aquatic ecosystems, especially due to artificial structures such as dams and bridge piers. This study investigates the swimming behavior differences between single and dual fish in the wake region behind a D-shaped obstacle, using Percocypris pingi as the experimental species. The results show that single fish efficiently utilize vortex energy through the Kármán gait, improving swimming efficiency, while the dual-fish group failed to maintain a stable Kármán gait, resulting in irregular swimming trajectories. However, the dual-fish group optimized wake utilization by maintaining a fore–aft linear alignment, improving swimming efficiency and resisting vortices. The conclusion indicates that mutual interference in group swimming affects swimming efficiency, with fish adjusting their swimming patterns to adapt to complex hydrodynamic conditions. By altering swimming formations, fish schools can adapt to the flow environment, offering new insights into the swimming behavior of fish and providing theoretical support for ecological conservation and hydropower project design.

## 1. Introduction

In recent decades, with the increasing impact of human activities on natural ecosystems, the changes to water flow caused by hydropower projects and river diversions have attracted widespread attention [1]. Water obstacles, particularly artificial structures such as dams, bridge piers, and reservoir inlets, often alter the flow velocity, flow pattern, and vortex structures, subsequently affecting the habitats and behavior patterns of aquatic organisms [2]. The presence of these water flow obstacles not only changes the natural flow of water but also forms complex vortices, wake regions, and turbulence zones [3]. The hydrodynamic characteristics of these regions have a profound impact on the swimming behavior of aquatic organisms, such as fish. In this context, understanding how fish adapt and adjust their swimming patterns in complex hydrodynamic environments is a significant topic in ecology, fluid dynamics, and aquatic biology [4,5,6,7].

The D-shaped obstacle, as a common water flow interference structure, can generate typical Kármán vortex streets and strong wake regions in the fluid [8,9,10]. This vortex flow structure causes uneven flow velocity, with significant variations in turbulence and vortex strength [11,12]. These changes not only influence the direction and velocity of the water flow but also create obstacles for fish swimming, leading to notable alterations in their swimming patterns [13,14]. Particularly in the wake region behind the D-shaped obstacle, fish may face challenges such as unstable water flow, large velocity gradients, and intense turbulence [15]. Existing research has mostly focused on the swimming behavior of fish in stable flow fields or simple flow environments, primarily studying how fish adjust their swimming posture, speed, and movement patterns to cope with fluid dynamics changes [16,17]. However, the performance of fish schools in complex water flow environments has received relatively less attention [18]. Schooling is a common behavior among many fish species in natural environments, especially when faced with complex water flows, predation threats, or reproductive needs [19,20,21]. Fish often cooperate within groups to enhance survival chances. However, group swimming, compared to individual swimming, faces additional challenges [22]. Fish in a group may interfere with each other, affecting the coordination of individual movements and the efficiency of energy use [23]. As a result, the swimming patterns in group swimming may differ from those in single-fish swimming, and in some cases, the swimming efficiency may even decrease [24].

The differences between single-fish and group swimming behaviors in complex water flow environments warrant in-depth investigation [25]. When swimming alone, fish can typically adjust their posture and trajectory to maximize the use of vortex energy in the water, thereby improving swimming efficiency [26]. However, in group swimming, the interactions between multiple individuals may lead to local instabilities in the fluid environment, and mutual interference may hinder synchronization between the individuals and the water flow, thus affecting the stability and efficiency of swimming [27]. For example, in vortex regions, multiple individuals may struggle to synchronize with the vortex frequency, as a single fish would, to reduce energy consumption. Instead, collisions and vortex interference may cause fluctuations in swimming trajectories and decrease efficiency. Therefore, researching the differences in behavior between single and dual fish in complex water flow conditions can provide valuable insights into how fish adjust their swimming strategies in groups and cope with water flow interference [28,29].

From a biomimetic perspective, studying fish swimming behavior in complex hydrodynamic environments holds not only ecological and conservation significance but also valuable implications for engineering applications. Over the course of evolution, fish have developed highly efficient propulsion mechanisms and energy-saving strategies that allow them to maintain stability and efficiency in unsteady flow fields [30]. These natural principles provide important theoretical foundations for the design of bionic underwater vehicles, robotic fish, and flow-control devices [31]. By analyzing how fish exploit vortex energy and fluid characteristics in wake and turbulent regions, researchers can develop more efficient biomimetic propulsion systems and adaptive control algorithms, thereby improving the maneuverability and energy efficiency of underwater robots operating in complex flow environments [32].

The *Percocypris pingi* (Jinsha bass carp) is a species native to the upper Yangtze River, known for its strong swimming abilities and adaptability to complex water flow environments [33]. However, due to habitat destruction and overfishing caused by human activities, the wild population of Jinsha bass carp has sharply declined and was listed as a nationally protected species in China [34]. Therefore, studying the swimming behavior of Jinsha bass carp in the wake region behind D-shaped obstacles, especially in vortex and wake areas, is of significant importance for the conservation of this species and the restoration of its habitat [35]. Furthermore, as a typical aquatic species, studying the swimming behavior of Jinsha bass carp not only has practical applications for species conservation but also provides valuable case studies for research on fish kinematics and fluid dynamics interactions [36].

The core objective of this study is to explore the differences in swimming behavior between single and dual fish in the wake region behind D-shaped obstacles. Specifically, the study aims to (1) simulate and analyze the hydrodynamic conditions of the water flow behind D-shaped obstacles, particularly the vortex structures and wake regions of the flow field. (2) Compare the kinematic parameters and morphological characteristics of single and dual fish under different water flow conditions. (3) Investigate the impact of interactions between individuals in school swimming on swimming patterns, analyzing how mutual interference and cooperation affect the swimming efficiency of fish. Through this study, we can not only deepen our understanding of how fish swim in complex hydrodynamic environments but also reveal the mechanisms of individual interactions in school swimming. The results of this study will not only have important implications for the conservation of species like the Jinsha bass carp but also provide theoretical support for the restoration of aquatic ecosystems, habitat rehabilitation, and the design of hydropower projects. At the same time, this research will contribute to the intersection of fish kinematics and fluid dynamics, enriching our understanding of how fish adapt to dynamic water flow conditions.

## 2. Materials and Methods

### 2.1. Experimental Fish

In this study, *Percocypris pingi* (*P. pingi*) was selected as the target experimental fish species. The Jinsha bass carp is mainly found in tributaries of the upper Yangtze River, including the Jinsha River, the Chishui River, and the Yalong River. Juvenile fish inhabit slow-flowing areas along the riverbanks, while adults are active in deep pools and swift currents. However, due to human activities such as hydropower development and overfishing, the wild population of the Jinsha bass carp has severely declined historically and was listed as a nationally protected species (Class II) in 2021. As an apex predator in the aquatic food chain of the upper Yangtze River, the recovery of this species directly reflects the ecological health of the waterbody. Through further research on the swimming behavior of this endangered species behind obstacles in the water, we aim to provide data support for the protection of its habitat and the restoration of the species. During this experiment, the water temperature and dissolved oxygen were maintained at 10–11 °C and 7.0–9.5 mg/L, respectively. The specific size information of the experimental fish was shown in Table 1. Two experimental conditions were implemented: S1 for single-fish trials, and T1 and T2 for dual-fish trials (T1: The first fish, T2: The second fish).

### 2.2. Experimental Setup

**Figure 1:**
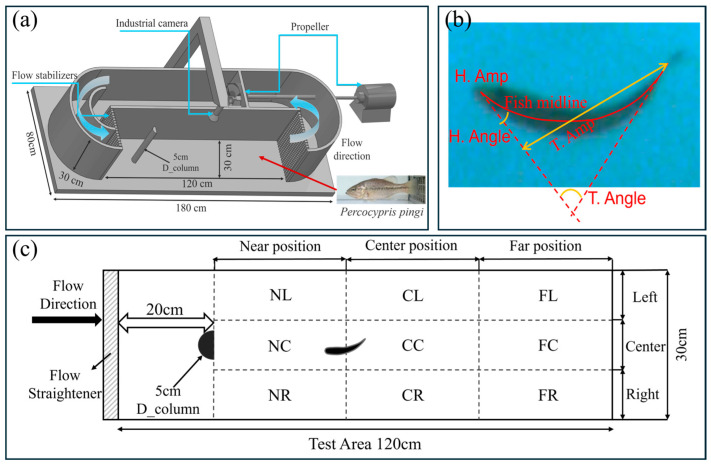
Experimental setup. (**a**) Daniel Loligo System equipped with D_column, industrial camera, propeller, and flow stabilizers; (**b**) schematic diagram of head and tail-beat angle and amplitude of fish body relative to fish midline; (**c**) testing region and schematic diagram of section behind D_column with zoning for positional analysis.

To simulate river channels with D-shaped obstacles under varying flow speeds, this study used a fish treadmill apparatus for physical experiments. As shown in Figure 1a, a variable-frequency propeller was employed to generate water flow moving counterclockwise at different speeds. Flow stabilizers were installed upstream of the experimental area to stabilize the water flow, reduce unnecessary turbulence, and enhance experimental accuracy. A flow direction device was also installed downstream to prevent the fish from being caught in the rotating blades, minimizing the risk of injury. An industrial camera was placed directly above the apparatus to record the swimming behavior and movement trajectories of *P. pingi*. The experimental area was divided into three main regions: Near position, Center position, and Far position, each further subdivided into three directional zones—Left, Center, and Right—creating a 120 cm × 30 cm test area as shown in Figure 1c. Specifically, the Near position includes NL, NC, and NR; the Center position includes CL, CC, and CR; the Far position includes FL, FC, and FR. Additionally, a D_column with a diameter of 5 cm was placed 20 cm upstream from the water inlet.

### 2.3. Main Indexes of Fish Behaviours

**Figure 2:**
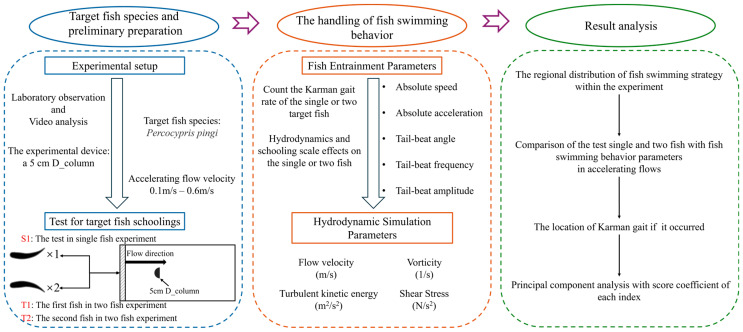
Flow chart of the methods used to study fish swimming behavior.

Swimming speed, swimming acceleration, tail beat frequency, tail beat angle, and tail beat amplitude are commonly used to assess the swimming performance and behavior of fish in a flow field. Figure 1b clearly demonstrates the definitions of three key morphological parameters of fish. Tail beat frequency refers to the number of complete tail beats per second, serving as an indicator of how fast the tail beats. Specifically, by observing the tail’s oscillation cycle in the diagram, the number of complete tail beats within a unit of time is calculated to determine the tail beat frequency. Tail beat angle is the angle between the tail and the fish’s body midline, reflecting the directionality and amplitude changes in the tail’s oscillation. The larger the angle, the greater the tail’s oscillation amplitude, which is typically associated with swimming speed and posture. Tail beat amplitude refers to the maximum vertical distance from the fish’s body midline to the tail’s tip, measuring the maximum deviation of the tail from the midline during a complete oscillation, reflecting the intensity of the tail’s movement. These parameters allow for a detailed analysis of fish swimming efficiency, tail movement control, and their ability to adapt to different flow environments. What’s more, to quantitatively identify the Kármán gait of fish in the wake of obstacles, this study integrates the coupling between fish body kinematics and flow field dynamics to comprehensively characterize the behavior. Specifically, when fish exhibit a Kármán gait, their bodies show a pronounced lateral deviation of the midline, the midline oscillates laterally within the wake channel relative to the main flow direction. At the same time, their tail-beat frequency is significantly lower than during active swimming, while the tail-beat amplitude increases correspondingly, indicating that the fish maintain a stable posture by passively responding to external vortex forces. Moreover, spectral analysis of tail-beat motions and vortex shedding reveals a synchronization in their dominant frequencies: the tail-beat frequency closely matches the vortex shedding frequency, demonstrating a coherent coupling between body movements and vortex structures. The specific research roadmap of this study is shown in Figure 2.

### 2.4. Hydrodynamic Simulation

#### 2.4.1. Model Setup

This numerical simulation uses the lattice Boltzmann method (LBM) to solve the fluid dynamics problem around a D-shaped cylinder, with efficient GPU computation carried out using the JAX library [37,38]. The target Reynolds number for the simulation is set to 30,000, with a characteristic length of 5 cm and kinematic viscosity of 0.01 cm^2^/s. The grid resolution of the computational domain is set based on the physical domain and computational resource constraints, ensuring numerical stability. To simulate turbulence and wall effects, the Smagorinsky subgrid-scale model (LES model) is used, with appropriate parameters set to capture the turbulent characteristics of the fluid. For boundary conditions, a no-slip boundary condition is applied to the cylinder surface, a velocity boundary condition is set at the inlet, and a convective boundary condition is applied at the outlet. Specifically, a hybrid wall function is used near the wall to adjust viscosity and ensure accurate simulation of the near-wall flow characteristics. For data collection and statistical analysis, multiple monitoring points are set in the cylinder’s wake region, and a statistical accumulator calculates the time-averaged velocity and turbulent kinetic energy (TKE) at these points. Simulation results are saved at each time step and periodically exported as CSV files for comparison with experimental data. To ensure continuity of the simulation, a checkpointing function is incorporated, periodically saving the simulation state and allowing the simulation to resume from the last saved state if interrupted. Simulation results are periodically exported in Tecplot format and visualized in real time to observe flow field evolution, while vorticity, velocity magnitude, and other advanced diagnostic indicators are calculated and recorded to help monitor the physical consistency and stability of the simulation process.

#### 2.4.2. Model Validation

To quantify the turbulence characteristics of the flow field, an Acoustic Doppler Velocimeter (ADV) was employed to measure the three-dimensional instantaneous velocity components, namely the streamwise (u), transverse (v), and vertical (w) velocities. The ADV provides high-frequency point measurements, enabling accurate capture of local velocity fluctuations. For each velocity component, the time-averaged mean velocity ($\bar{u}$, $\bar{v}$, $\bar{w}$) was first computed, and the fluctuating components were obtained as:

(1) $$\begin{array}{c} u^{\prime }=u-\bar{u},v^{\prime }=v-\bar{v},w^{\prime }=w-\bar{w} \end{array}$$

The turbulent kinetic energy (TKE) was then estimated using the following expression:

(2) $$\begin{array}{c} k=\frac{1}{2}\left(\bar{{u^{\prime }}^{2}}+\bar{{v^{\prime }}^{2}}+\bar{{w^{\prime }}^{2}}\right) \end{array}$$

where $\bar{{u^{\prime }}^{2}}$, $\bar{{v^{\prime }}^{2}}$, and $\bar{{w^{\prime }}^{2}}$ represent the time-averaged variances of the velocity fluctuations in each direction. This approach is widely adopted in experimental fluid mechanics to assess local turbulence intensity based on point velocity measurements.

**Figure 3:**
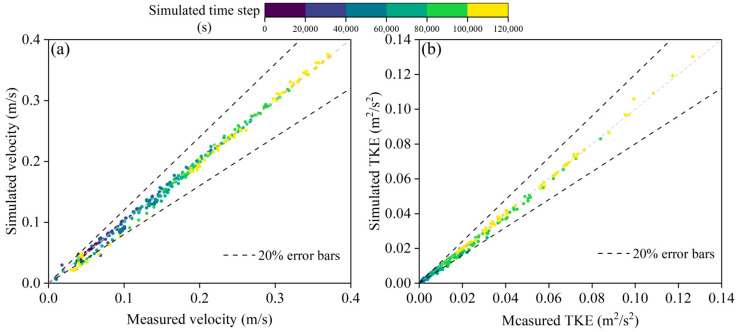
Figures show comparison of measured and numerically simulated flow velocity (**a**) and turbulent kinetic energy results (**b**).

**Figure 4:**
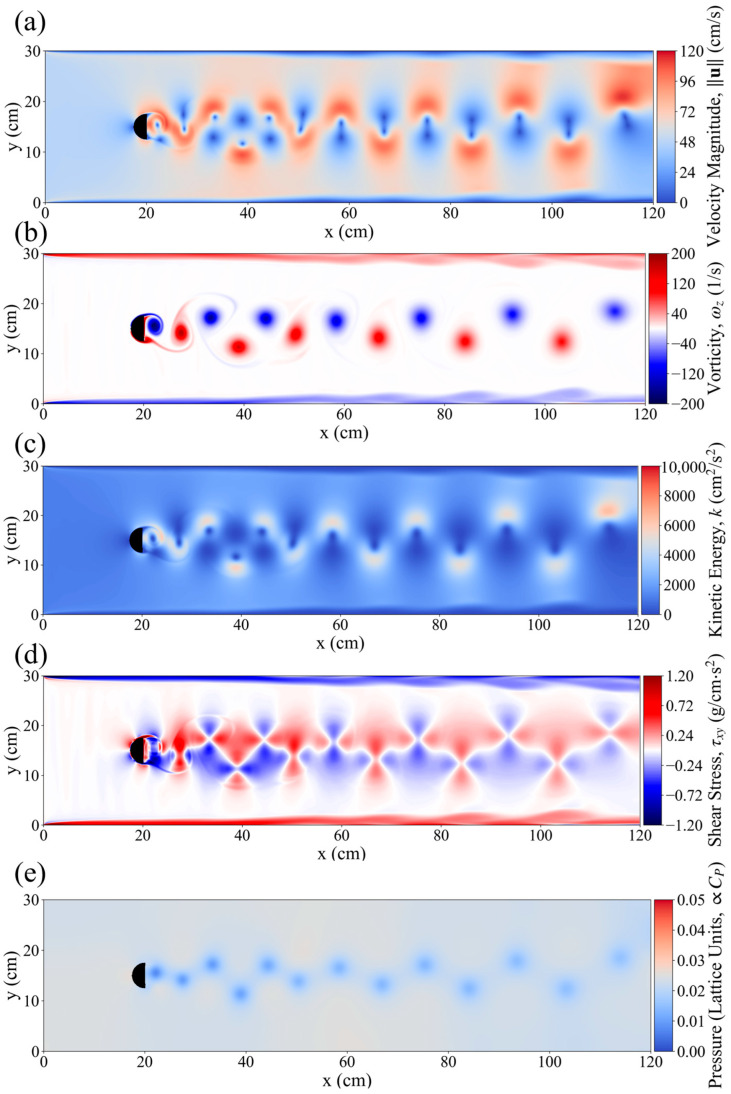
(**a**) Velocity magnitude, (**b**) vorticity, (**c**) kinetic energy (KE), (**d**) sheer stress and (**e**) pressure around the flow field downstream of D_column, under inlet velocity of 0.6 cm/s.

To validate the effectiveness of the simulation results, this study compared the measured flow velocity and turbulent kinetic energy (TKE) from the experimental setup with the simulation results (Figure 3). The measurement points were evenly distributed throughout the experimental setup, with a total of 40 measurement points. An Acoustic Doppler Velocimeter (ADV) was used to measure the flow velocity and TKE, with a measurement range of 0.01–10.00 m/s and an error of 1.0%. The comparison of the measured results with the simulated flow velocity and TKE is shown in Figure 4. The relative error range for flow velocity was approximately 1.23% to 21.78%, with an average relative error of 6.45%. The relative error range for TKE was approximately 2.34% to 20.25%, with an average relative error of 3.13%. Overall, the simulation results of this study are able to reflect the hydrodynamic conditions in the experimental setup reasonably well.

## 3. Result

### 3.1. Hydrodynamic Status

This study simulated the hydrodynamic conditions in a flume with a D-shaped obstacle under a flow velocity of 0.6 m/s. Figure 4 illustrates the distributions of pressure, shear stress, turbulent kinetic energy, vorticity, and velocity gradient in the wake region. In the wake area behind the D-shaped obstacle, two alternating Kármán vortex streets emerged, with completely opposite rotation directions. The maximum intensity occurred at 20–40 cm downstream, reaching a vorticity of 200 1/s, after which the vorticity gradually decreased. In addition, the distributions of velocity gradient and turbulent kinetic energy exhibited similar trends. Specifically, in regions near the vortices, the velocity gradient and turbulent kinetic energy surged, reaching 96–120 cm/s and 5500 cm^2^/s^2^, respectively. Due to the reverse rotation and vertical alternating arrangement of the vortices, the high-velocity gradient and high-turbulent kinetic energy regions presented an S-shaped pattern, interwoven with low-velocity gradient areas (Figure 3a,b). Correspondingly, the shear stress field was closely related to the vortex street distribution, showing an alternating pattern of positive and negative values, with a peak value of up to 1.2 g/cm^2^. In contrast, the pressure field fluctuations were relatively weak, with significant changes primarily concentrated in the core regions of downstream vortices, appearing as point-like distributions without disturbing the areas between vortices. The hydrodynamic field behind the D-shaped obstacle exhibited typical Kármán vortex street characteristics. This periodic vortex shedding structure dominated the downstream flow state, not only leading to an S-shaped interwoven distribution of turbulent kinetic energy and velocity gradient but also forming strong alternating shear stress regions, which had a significant impact on the energy transport in the entire flow field as well as the subsequent swimming behavior of *P. pingi*.

### 3.2. Fish Swimming Kinematics

**Figure 5:**
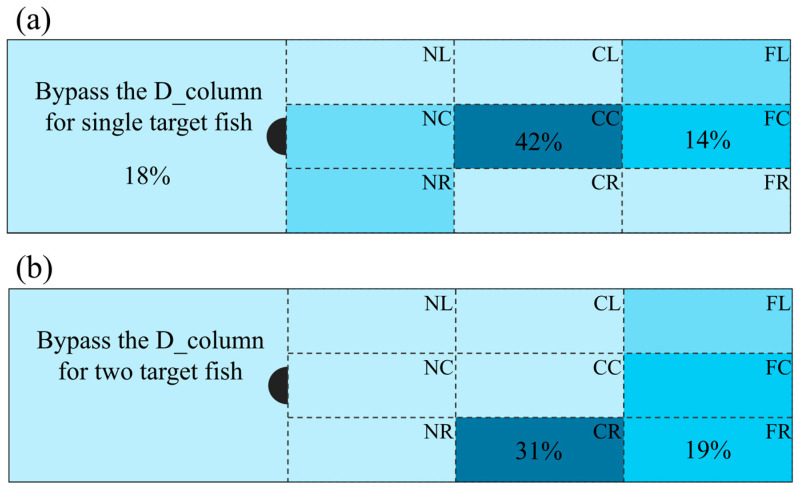
Swimming preference of fish around D_column under flow velocity of 0.3–0.6 cm/s: (**a**) for single target fish, (**b**) for two target fish. The proportion of time occupied in each Sub-region is indicated by the shade of blue, with a deeper shade corresponding to a higher proportion.

All fish kinematic parameters were calculated from experimental videos. Figure 5 shows the swimming area preference of *P. pingi* at flow velocities of 0.3–0.6 m/s. Under the single fish condition, *P. pingi* typically swims in the central region of the downstream wake zone beneath the D-shaped column (CC and FC), and at 15–20 cm, it enters the Kármán gait, occupying 42% of the test time. In contrast, under the dual fish condition, it was found that *P. pingi* does not swim directly behind the D-shaped column but tends to swim in the boundary regions of the test area (CR and FR), occupying 50% of the test time. Notably, in the dual fish experiment, *P. pingi* was unable to maintain a continuous and long-lasting Kármán gait, with the two fish mostly swimming in a front-and-rear linear arrangement. This arrangement may be a result of mutual cooperation between the group to resist vortices and avoid collisions.

**Figure 6:**
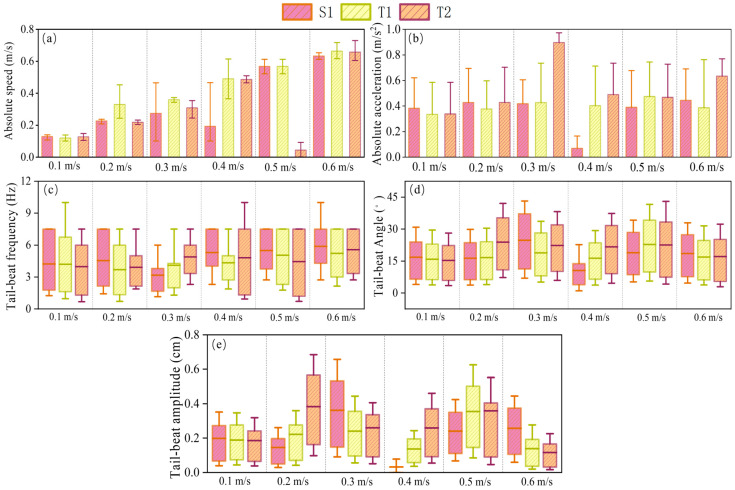
Kinematic parameters of fish swimming under different flow velocities: (**a**) absolute speed, (**b**) absolute acceleration, (**c**) tail-beat frequency, (**d**) tail-beat angle, (**e**) tail-beat amplitude, for groups S1, T1, and T2.

Figure 6 demonstrates the effect of different fish quantities on the swimming kinematic characteristics of *P. pingi* at flow velocities ranging from 0.1 m/s to 0.6 m/s, including absolute swimming speed, acceleration, tail beat frequency, tail beat angle, and tail beat amplitude. The swimming speed of S1 was generally higher under all flow conditions and increased with increasing flow velocity. In contrast, the swimming speed of T1 and T2 fluctuated more, with T2 showing larger fluctuations at higher flow velocities, such as a sudden drop to 0.024 m/s at 0.5 m/s. The swimming speed of T1 remained relatively stable. Regarding acceleration, S1 experienced a sharp decline in acceleration to only 0.078 m/s^2^ at a flow velocity of 0.4 m/s, with no significant changes at other flow velocities, remaining around 0.4 m/s^2^. However, T2’s acceleration increased with flow velocity, being much higher than the other two groups at 0.3 m/s and 0.6 m/s, at 0.91 m/s^2^ and 0.62 m/s^2^, respectively. The tail beat frequency in the S1 experiment remained between 3–7 Hz, slightly increasing with flow velocity, while T1 and T2 showed larger fluctuations. The trends of tail beat angle and amplitude were similar. Notably, at 0.3 m/s, the tail beat frequency of S1 was much lower than that of T1 and T2, but its tail beat angle and amplitude were significantly higher, which may be due to the fact that *P. pingi* enters the Kármán gait in the single fish condition, while under the dual fish condition, *P. pingi* cannot maintain the Kármán gait for an extended period.

**Figure 7:**
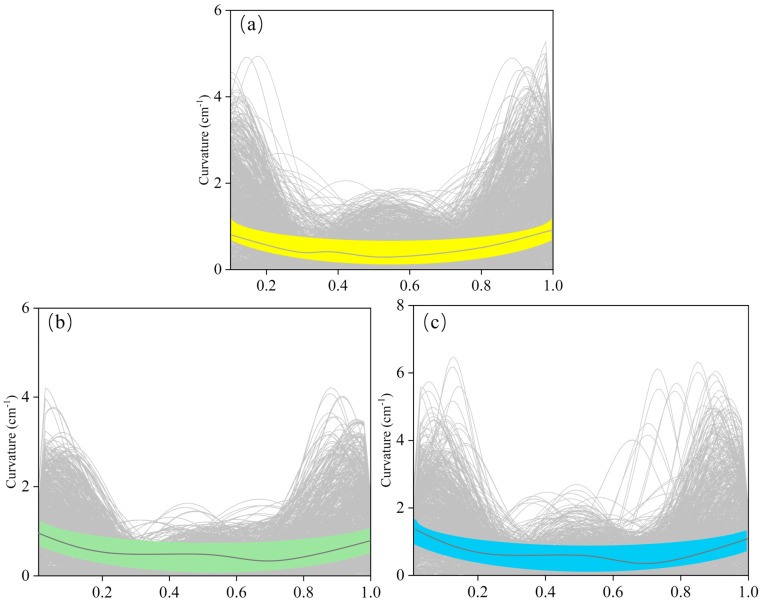
Variation in fish body curvature in the flow field around D_column for groups (**a**) S1, (**b**) T1, and (**c**) T2, the colored band shows the 90% confidence band.

Figure 7 shows the effect of different conditions on fish body curvature, with curvature ranging from 0 to 1.2 cm^−1^. Overall, the curvature changes at the head and tail were more pronounced, with T2 showing relatively stable curvature at all positions with little variation. Notably, the curvature of S1 was generally lower than that of the T groups, and under the same condition, the average curvature of T2 was higher than T1 by approximately 0.9 cm^−1^ (Table 2).

### 3.3. Fish Kinematics Under Kármán Gait

**Figure 8:**
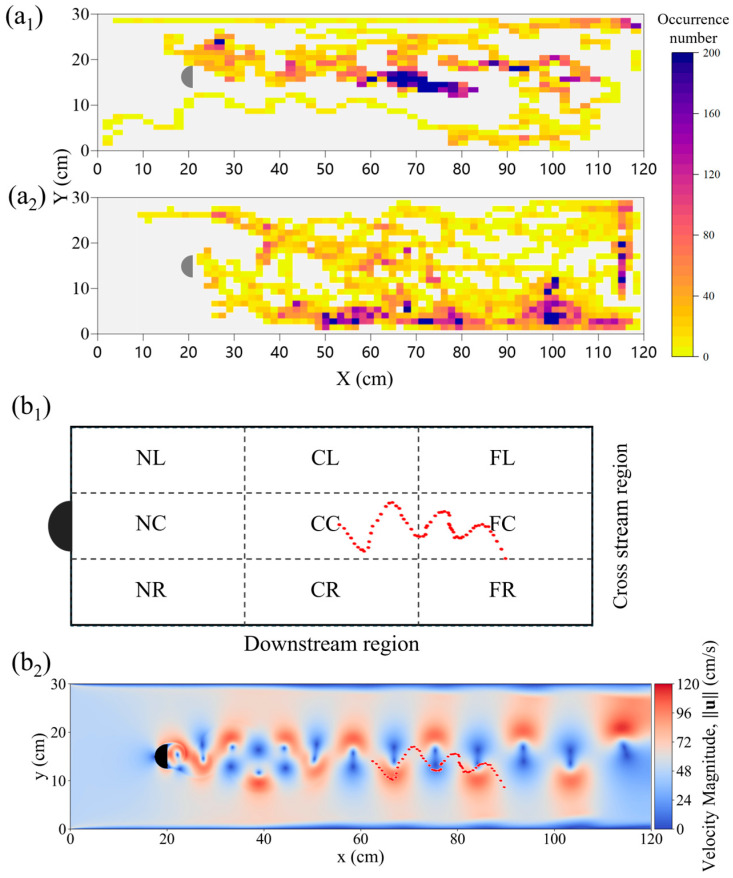
Fish movement characteristics under two different conditions: Heat maps of occurrence number for fish groups: (**a_1_**) S1, (**a_2_**) T1 + T2, at an inlet velocity of 0.1–0.6 m/s; Trajectory of a single fish in condition S1: (**b_1_**) within the zoned test area, the red trajectory represents a typical Kármán gait of the fish S1 in the wake region. (**b_2_**) superimposed on the flow velocity magnitude field, at an inlet velocity of 0.6 m/s.

**Figure 9:**
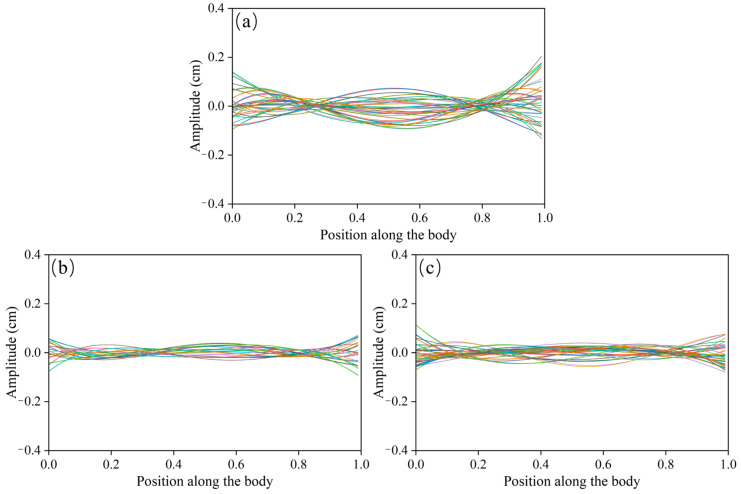
Variation in fish body midline amplitude under different fish groups and flow condition induced by D_column at 0.6 m/s: (**a**) S1; (**b**) T1; (**c**) T2, the different colors indicate the phase shift of the fish’s centerline oscillation.

This study further analyzed the kinematic and morphological characteristics of *P. pingi* under the Kármán gait. Figure 8a_1_,a_2_ show the movement trajectories and distributions of *P. pingi* under two different conditions. In the S1 group, *P. pingi* tended to stay near the center of the flume, 60–80 cm downstream, with an occurrence number as high as 200, demonstrating significant spatial aggregation. However, in the experiments with two fish, *P. pingi* was more inclined to move near the flume boundaries, with a wider activity range covering the entire wake region. Since the *P. pingi* in the T group failed to maintain a stable Kármán gait, Figure 8b_1_,b_2_ present the trajectories of *P. pingi* in the S1 group under a flow velocity of 0.6 m/s while performing the Kármán gait. The Kármán gait mainly appeared in the CC and FC regions downstream of the D-shaped obstacle, 15–25 cm away from the obstacle, corresponding to 3–5 times the obstacle’s diameter. This region coincided with the spatial location where vortex shedding and velocity gradients were most pronounced. Considering the hydrodynamic characteristics, the trajectories of *P. pingi* under the Kármán gait resembled a sinusoidal wave, but the amplitudes and positions of the peaks and troughs shifted and adjusted with local vortex variations, exhibiting an upstream swimming pattern that took advantage of the energy from vortices and high velocity gradients. Figure 9 illustrates the midline fluctuations of *P. pingi* under different conditions. In the S1 single-fish experiment group (Figure 9a), the midline of *P. pingi* showed a clear lateral deviation, and the overall waveform during the test period was closer to a standing wave pattern. In particular, the oscillation amplitude of the head and tail was significantly enhanced, which is closely related to the ability of the individual to maintain the Kármán gait for a prolonged period under the S1 condition. The maintenance of the Kármán gait enabled the fish body to lock in with the vortex shedding frequency of the flow field, resulting in a more regular and concentrated midline fluctuation, while larger tail oscillations facilitated efficient energy utilization. In contrast, in the T group dual-fish experiment (Figure 9b,c), due to mutual interference between individuals and insufficient flow field stability, *P. pingi* was unable to sustain the Kármán gait. As shown in the figures, the midline variations in the two fish during the test period did not exhibit obvious lateral deviations; instead, they presented a mixed waveform combining standing and traveling waves. This waveform indicates that the fish body continuously attempted to exploit the hydrodynamic advantages brought by vortices in the flow field; however, without forming stable wake synchronization, the movement trajectories displayed greater variability and more dispersed energy distribution. Overall, the midline fluctuations of the S1 experimental group exhibited concentration and regularity, whereas those of the T group experiment displayed dispersion and diversity.

## 4. Discussion

This study explores the swimming behavior of fish in complex hydrodynamic environments, with a focus on the differences between single-fish and dual-fish behaviors in the wake of a D-shaped obstacle. The research highlights the persistence of the Kármán gait under different experimental conditions, particularly contrasting single-fish and group scenarios.

### 4.1. Relationship Between Hydrodynamic Environment and Fish Behavior

The influence of hydrodynamic conditions on fish swimming behavior is fully demonstrated in this study. The results show that in the wake of a D-shaped obstacle, the formation of a Kármán vortex street creates a periodic flow field. Fish can lock onto the vortex frequency to maintain the Kármán gait. Consistent with the previous findings [39,40], single fish can effectively harness the energy provided by vortices for efficient swimming, achieving greater stability by sustaining the Kármán gait. In the single-fish experiment (Group S1), *P. pingi* successfully maintained a distinct Kármán gait and swam in the central wake region. This allowed the fish to synchronize with the vortex frequency in the flow field, thereby maximizing energy utilization. In contrast, in the dual-fish experiments (Group T), although both fish swam within the flow field, they did not establish a stable Kármán gait. Instead, they adopted a linear fore–aft arrangement and failed to sustain frequency locking with the vortices over time. This starkly contrasts the regular swimming observed under single-fish conditions. In the dual-fish case, *P. pingi* tended to swim toward the wake boundaries, suggesting that mutual interference and flow instability in group conditions limited the persistence of the Kármán gait.

### 4.2. Kármán Gait and Swimming Efficiency

Under single-fish conditions, *P. pingi* successfully maintained the Kármán gait, demonstrating that fish in stable vortex flows can synchronize with vortex frequency to achieve efficient swimming. Liao pointed out that the Kármán gait helps fish reduce energy consumption while increasing swimming speed [41]. The results of Grpup S1 showed that *P. pingi* maintained relatively high swimming speeds within the 0.3–0.6 m/s flow range, with tail beat amplitude and frequency displaying clear regularity—fully showcasing the efficiency of the Kármán gait. However, under dual-fish conditions, *P. pingi* failed to sustain a stable Kármán gait and instead displayed considerable instability. Particularly in Groups T1 and T2, the two fish exhibited large fluctuations in swimming speed, with sharp declines in performance. Unlike the single-fish case, they were unable to efficiently harness vortex energy. This phenomenon can be attributed to mutual interference between individuals, preventing synchronized Kármán gait formation. The fish only maintained a fore–aft linear alignment and did not achieve the efficient swimming pattern observed in single fish. This result suggests that group swimming may lead to the breakdown of the Kármán gait due to inter-individual effects, thereby reducing overall swimming efficiency.

### 4.3. Mutual Interference in Group Swimming

The impact of mutual interference on swimming efficiency in group swimming is a key finding of this study. In single-fish experiments, *P. pingi* was able to stably maintain the Kármán gait, efficiently exploiting vortex energy. However, in the dual-fish experiments, *P. pingi* did not adopt the traditional Kármán gait but instead showed a fore–aft linear alignment pattern, characterized by greater motion fluctuations and energy dispersion. This study argues that the absence of the Kármán gait in dual-fish groups was not simply due to “mutual interference.” Rather, the two fish adopted a cooperative fore–aft configuration that altered the local hydrodynamic conditions. In this arrangement, the fish reduced drag between individuals while optimizing wake utilization, thereby improving overall swimming efficiency. Although this configuration did not maintain the Kármán gait, it still exploited vortex energy to enhance swimming efficiency [42]. Thus, the findings suggest that in dual-fish swimming, mutual cooperation and flow-field modifications may lead to swimming modes even more efficient than the traditional Kármán gait. Although inter-individual interference typically disrupts the gait, fish groups can sometimes adapt by changing their swimming formation to further optimize efficiency [43].

### 4.4. Relationship Between Midline Oscillation and Swimming Modes

In the single-fish experiments, *P. pingi* exhibited pronounced lateral midline oscillations resembling a standing wave pattern. This indicates that the fish was able to maintain the Kármán gait, with a highly regular and concentrated trajectory. Under these conditions, synchronization with vortex frequency allowed the fish to effectively harness vortex energy to improve efficiency. The stability of the Kármán gait ensured coordination between head and tail movements, producing a stable trajectory with smaller oscillations and enhancing swimming stability and efficiency [44]. In contrast, under dual-fish conditions, *P. pingi*’s midline oscillations showed a mixed waveform, containing both standing and traveling wave components. This suggests that the swimming trajectories became less regular, with greater fluctuations and energy dispersion. Without a stable Kármán gait, the fish could not remain synchronized with vortex frequency, leading to irregular trajectories. This phenomenon is closely linked to mutual interference and flow instability, further confirming that unsynchronized swimming reduces efficiency. In dual-fish experiments, interactions often led to strongly asynchronous trajectories. Although the fish attempted to exploit vortex energy, interference between individuals prevented synchronization, resulting in more dispersed and unstable paths. Compared with the single-fish case, midline oscillations in the dual-fish condition displayed greater energy dispersion and larger amplitudes. This highlights the challenges of group swimming: while single fish can achieve efficient Kármán gait swimming, multiple fish swimming together may experience asynchronous interactions that reduce stability and efficiency. In natural environments, fish schools facing complex flow conditions and mutual interference may adopt more flexible, though less efficient, swimming modes instead of relying on stable Kármán gaits. Moreover, such irregular oscillations and mixed standing–traveling waveforms may be associated with escape responses or adaptive changes to flow disturbances. In group swimming, when synchronization fails, individuals may engage in more frequent adjustments to cope with turbulence and mutual interference, leading to greater motion fluctuations and energy dispersion.

## 5. Conclusions

This study selected *P. pingi* as the target fish species to investigate its swimming behavior in the wake of a D-shaped column, comparing the differences between single-fish and dual-fish conditions. The results showed that under single-fish conditions, *P. pingi* efficiently utilized vortex energy through the Kármán gait at 3–5 times the obstacle radius downstream, improving swimming efficiency. In contrast, the dual-fish group failed to maintain a stable Kármán gait, leading to irregular swimming trajectories. Nevertheless, the dual-fish group optimized wake utilization by maintaining a fore–aft linear alignment in the boundary regions of the tank, improving swimming efficiency and resisting vortices. Additionally, this study found that the midline of fish in the S1 group exhibited a clear standing wave pattern, while the midline of fish in the dual-fish group showed a combination of standing and traveling waves. This result suggests that synchronization issues in group swimming significantly affect swimming efficiency, and fish may adjust their swimming patterns to adapt to different flow conditions in complex hydrodynamic environments.

## Figures and Tables

**Table 1:** Description of the experimental trials.

Flow Condition	Velocity at Inlet Range (m/s)	Fish Body Length (cm)	Fish Body Weight (g)	Time Duration (min)	Fish Used Number in Experiment
S1	0.1–0.6	14.59 ± 0.92	18.44 ± 4.86	26.7 ± 4.8	10
T1 + T2	0.1–0.6	13.81 ± 1.08	18.03 ± 3.81	28.5 ± 3.1	10

**Table 2:** Positional curvature of the fish body across various body regions.

Flow Condition	Positional Parameters and Curvature (cm^−1^) of the Fish Body				
	0.00	0.25	0.50	0.75	1.00
S1	0.488554	0.756028	0.65355	1.053047	0.667764
T1	0.120473	0.904363	1.014549	0.771405	0.533283
T2	0.800117	0.8680525	1.1014916	0.9915422	0.991542

## Data Availability

Data are contained within the article.

## References

[1] Xie O., Sun Z., Shen C. (2023). A study on flow-field characteristics of a self-propelled robot fish approaching static obstacles based on artificial lateral line. Bioinspir. Biomim..

[2] Zhang J., Tang R., Yao W., Zheng T., Pan D., Wang H. (2024). Hydraulic study of bionic robotic fish swimming downstream of submerged obstacles. Ocean Eng..

[3] Xie Q., Wang L., Yang S., Hu J., Li W., Yang W., Zhang X., Zhang P. (2025). Collective behavior and upstream tactics of schooling fish in an obstacle environment. Environ. Biol. Fishes.

[4] Cassan L., Miranda F.C., Laurens P. (2023). Hydraulic Resistance in Rock-Ramp Fish Passes for Various Shapes of Macro-roughness. J. Hydraul. Eng.-ASCE.

[5] Nyqvist D., Tarena F., Candiotto A., Comoglio C. (2024). Individual activity levels and presence of conspecifics affect fish passage rates over an in-flume barrier. Ecol. Freshw. Fish.

[6] Potrich D., Orsini C., Stancher G., Baratti G., Sovrano V.A. (2024). A Comparison of Detour Behaviors in Some Marine and Freshwater Fish Species. Animals.

[7] Wang F., Kobina F. (2025). The influence of geological factors and transmission fluids on the exploitation of reservoir geothermal resources: Factor discussion and mechanism analysis. Reserv. Sci..

[8] Ma Q., Wang J. (2025). Numerical investigation of schooling arrangement and phase differences on the hydrodynamic performance of fish swimming side-by-side behind a D-cylinder. Phys. Fluids.

[9] Mao X., Wang J., Mao X., Deng J. (2025). A hybrid numerical model for the collective motion of fish groups. J. Fluid Mech..

[10] Wang H., Yuan S., Tang H., Gualtieri C., Ling Y. (2025). Hydrodynamic performance of swimming fish in the wake region of a semi-cylinder. Ocean Eng..

[11] Akanyeti O., Liao J.C. (2013). The effect of flow speed and body size on Kármán gait kinematics in rainbow trout. J. Exp. Biol..

[12] Zheng X., Yu W., Cao C., Xi Y., Zhao Y., Li D., Ouyang L., Wang R., Liu Y., Yu F. (2025). Effects of vortex flows induced by four obstacles on swimming behaviors of juvenile *Onychostoma sima*. J. Ecohydraul..

[13] Liao J.C., Akanyeti O. (2017). Fish Swimming in a Kármán Vortex Street: Kinematics, Sensory Biology and Energetics. Mar. Technol. Soc. J..

[14] Wu J., Ansari U. (2025). From CO_2_ Sequestration to Hydrogen Storage: Further Utilization of Depleted Gas Reservoirs. Reserv. Sci..

[15] Yan L., Chang X., Wang N., Tian R., Zhang L., Liu W. (2021). Computational analysis of fluid-structure interaction in case of fish swimming in the vortex street. J. Hydrodyn..

[16] Harvey S.T., Muhawenimana V., Müller S., Wilson C.A.M.E., Denissenko P. (2022). An inertial mechanism behind dynamic station holding by fish swinging in a vortex street. Sci. Rep..

[17] Shi X., Hu Q., Zhang T., Li S., Zeng Y. (2024). Research on hydrodynamic performance of 2D undulating fin in the wake of a semi-cylinder. Ocean Eng..

[18] Connor J., Joordens M., Champion B. (2023). Fish-inspired robotic algorithm: Mimicking behaviour and communication of schooling fish. Bioinspir. Biomim..

[19] Kasumyan O., Pavlov D.S. (2023). Mechanisms of Schooling Behavior of Fish. J. Ichthyol..

[20] Kasumyan A.O., Pavlov D.S. (2023). Hydrodynamics and Energetics of Schooling Swimming and Migration of Schooling Fish. J. Ichthyol..

[21] Kelly J., Pan Y., Menzer A., Dong H. (2023). Hydrodynamics of body-body interactions in dense synchronous elongated fish schools. Phys. Fluids.

[22] Wei Y., Ji L., An D. (2025). Review on Quantitative Methods of Fish School Behaviors. Rev. Aquac..

[23] Larrieu R., Quilliet C., Dupont A., Peyla P. (2021). Collective orientation of an immobile fish school and effect on rheotaxis. Phys. Rev. E.

[24] Yoshida K., Ogata Y., Hirai S., Hosotani K. (2023). Numerical study of the correlation between fish school arrangement and propulsive performance. Artif. Life Robot..

[25] Li X., Gu J., Su Z., Yao Z. (2021). Hydrodynamic analysis of fish schools arranged in the vertical plane. Phys. Fluids.

[26] Ligman M.G., Lund J., Fürth M. (2023). A comprehensive review of hydrodynamic studies on fish schooling. Bioinspir. Biomim..

[27] Zhou J., Seo J.-H., Mittal R. (2025). Effect of hydrodynamic wakes in dynamical models of large-scale fish schools. Phys. Fluids.

[28] Peterson A.N., Swanson N., McHenry M.J. (2024). Fish communicate with water flow to enhance a school’s social network. Exp. Biol..

[29] Song J., Li Y., Xiao Y., Wang C., Zhong Y., Yin L. (2024). Delayed action leads to faster turning of fish by interaction with neighbor. Phys. Fluids.

[30] Zhang Z., Wang Q., Zhang S. (2024). Review of Computational Fluid Dynamics Analysis in Biomimetic Applications for Underwater Vehicles. Biomimetics.

[31] Abbaszadeh S., Leidhold R., Hoerner S. (2022). A Design Concept and Kinematic Model for a Soft Aquatic Robot with Complex Bio-mimicking Motion. J. Bionic Eng..

[32] van den Berg S.C., Scharff R.B., Rusák Z., Wu J. (2022). OpenFish: Biomimetic design of a soft robotic fish for high speed locomotion. HardwareX.

[33] Li L., Li M., Zeng R., Deng L., Yang K., Song Z. (2023). High resistance to *Ichthyophthirius multifiliis* in *Percocypris pingi*, an endemic fish in upper Yangtze River. Aquaculture.

[34] He Z., Li C., Gao K., Zheng X., Wang X., Wang H., Chen Q., Tang Z., Zhang M., Yang D. (2024). The whole chromosome-level genome provides resources and insights into the endangered fish *Percocypris pingi* evolution and conservation. BMC Genom..

[35] Lacey R., van Leeuwen J., Munoz R.M. (2012). Influence of bluff body wake structure on fish preferred holding locations. River Flow 2012, Volumes 1 and 2.

[36] Cai L., Hou Y., Katopodis C., He D., Johnson D., Zhang P. (2019). Rheotaxis and swimming performance of Perch-barbel (*Percocypris pingi*, Tchang, 1930) and application to design of fishway entrances. Ecol. Eng..

[37] Cheng Y., Zhang H. (2010). Immersed boundary method and lattice Boltzmann method coupled FSI simulation of mitral leaflet flow. Comput. Fluids.

[38] Yu Z., Fan L.-S. (2010). Lattice Boltzmann method for simulating particle-fluid interactions. Particuology.

[39] Liao J.C. (2007). A review of fish swimming mechanics and behaviour in altered flows. Philos. Trans. R. Soc. B-Biol. Sci..

[40] Maia A., Sheltzer A.P., Tytell E.D. (2015). Streamwise vortices destabilize swimming bluegill sunfish (*Lepomis macrochirus*). J. Exp. Biol..

[41] Liao J.C., Rajeev E., Canestrelli A., Ray B. (2021). Flooded forests in flow; trout exploit wakes behind multi-cylinder arrays. Integr. Comp. Biol..

[42] Burgerhout E., Tudorache C., Brittijn S.A., Palstra A.P., Dirks R.P., Thillart G.E.v.D. (2013). Schooling reduces energy consumption in swimming male European eels, *Anguilla anguilla* L.. J. Exp. Mar. Biol. Ecol..

[43] Yang F., Zeng Y. (2024). Collective swimming pattern and synchronization of fish pairs (*Gobiocypris rarus*) in response to flow with different velocities. J. Fish Biol..

[44] Liao J.C. (2004). Neuromuscular control of trout swimming in a vortex street: Implications for energy economy during the Karman gait. J. Exp. Biol..

